# Disrupted Spatial Organization of Cued Exogenous Attention Persists Into Adulthood in Developmental Dyslexia

**DOI:** 10.3389/fpsyg.2021.769237

**Published:** 2021-11-15

**Authors:** Ana Pina Rodrigues, Miguel Castelo-Branco, Marieke van Asselen

**Affiliations:** ^1^Coimbra Institute for Biomedical Imaging and Translational Research (CIBIT), Institute of Nuclear Sciences Applied to Health, University of Coimbra, Coimbra, Portugal; ^2^Faculty of Medicine, University of Coimbra, Coimbra, Portugal

**Keywords:** dyslexia, exogenous attention, visual eccentricity, reaction time, cueing

## Abstract

**Purpose:** Abnormal exogenous attention orienting and diffused spatial distribution of attention have been associated with reading impairment in children with developmental dyslexia. However, studies in adults have failed to replicate such relationships. The goal of the present study was to address this issue by assessing exogenous visual attention and its peripheral spatial distribution in adults with developmental dyslexia.

**Methods:** We measured response times, accuracy and eye movements of 18 dyslexics and 19 typical readers in a cued discrimination paradigm, in which stimuli were presented at different peripheral eccentricities.

**Results:** Results showed that adults with developmental dyslexia were slower that controls in using their mechanisms of exogenous attention orienting. Moreover, we found that while controls became slower with the increase of eccentricity, dyslexics showed an abnormal inflection at 10° as well as similar response times at the most distant eccentricities. Finally, dyslexics show attentional facilitation deficits above 12° of eccentricity, suggesting an attentional engagement deficit at far periphery.

**Conclusion:** Taken together, our findings indicate that, in dyslexia, the temporal deficits in orientation of attention and its abnormal peripheral spatial distribution are not restricted to childhood and persist into adulthood. Our results are, therefore, consistent with the hypothesis that the neural network underlying selective spatial attention is disrupted in dyslexia.

## Introduction

Developmental dyslexia (DD) is characterized by a reading impairment, despite normal intelligence and adequate reading instruction. Although phonological processing deficits are well established as core deficits in DD (Snowling, 1981; Ziegler and Goswami, 2005), it has been suggested that attentional impairments may also contribute to the pathophysiology of this condition (Cestnick and Coltheart, 1999; Vidyasagar, 1999, 2019; Hari and Renvall, 2001; Facoetti et al., 2005, 2006; Bosse et al., 2007; Vidyasagar and Pammer, 2010; Pina Rodrigues et al., 2017a). Accordingly, several types of attention deficits have been reported in DD: narrowed visual attentional window and reduced visual attention span (Bosse et al., 2007); stronger effects of crowding (Bouma and Legein, 1977; Spinelli et al., 2002; Pernet et al., 2006; Martelli et al., 2009; Moores et al., 2011; Callens et al., 2013); noise exclusion deficits (Sperling et al., 2005, 2006; Pina Rodrigues et al., 2017a); and, particularly relevant for this study, abnormal spatial distribution of attention (Facoetti and Turatto, 2000; Facoetti and Molteni, 2001) and impaired attention orienting (Brannan and Williams, 1987; Facoetti et al., 2000b, 2003a, 2006; Facoetti and Molteni, 2001; Hari and Renvall, 2001; Kinsey et al., 2004; Valdois et al., 2004; Roach and Hogben, 2008; Vidyasagar and Pammer, 2010; Franceschini et al., 2012; Gabrieli and Norton, 2012). Moreover, it has been shown that prereading visuo-attentional skills can predict reading impairments (Franceschini et al., 2012; Carroll et al., 2016; Valdois et al., 2019) and that attentional training is able to improve reading in dyslexics (Franceschini et al., 2013), suggesting a causal link between attentional deficits and reading impairments.

Fluent reading requires precise and rapid selection of relevant stimuli among distractors (Bouma, 1970; Bouma and Legein, 1977; Reynolds and Besner, 2006), which critically requires efficient orientation of attention (Cestnick and Coltheart, 1999; Vidyasagar, 1999; Facoetti et al., 2006; Perry et al., 2007; Vidyasagar and Pammer, 2010). In particular, the orientation onto each sublexical unit is crucial for graphemic parsing, defined as the process determining the graphemic elements of a word, which, according to computational models of reading, precede spelling-to-sound conversion mechanisms (McCandliss et al., 2003; Whitney and Cornelissen, 2005; Perry et al., 2007). Indeed, before the application of the grapheme-to-phoneme correspondences, graphemes have to be accurately selected through rapid serial attentional orienting. This mechanism allows the selective processing of relevant letter-to-speech sound correspondence while suppressing the irrelevant ones.

Spatial orientation of attention can be voluntary, *via* a mechanism known as endogenous attention, or automatic, stimulus-driven, termed exogenous attention (Fuller et al., 2008). These two systems are also labeled as sustained (endogenous) and transient (exogenous) due to the difference in their processing time-courses. Whereas the effects of endogenous attention require few hundred milliseconds to fully develop and can be maintained with effort, exogenous attention peaks within 100 to 120 ms and diminishes rapidly thereafter (Nakayama and MacKeben, 1989; Cheal and Lyon, 1991).

It is worth pointing out that the attentional orienting system is anatomically based in the parietal dorsal stream, which in turn, has strong input from the magnocellular system (Gori and Facoetti, 2015). Several studies have shown temporal deficits in DD often suggested to be associated with magnocellular dysfunction (Livingstone et al., 1991; Cornelissen et al., 1995; Stein and Walsh, 1997; Iles et al., 2000; Talcott et al., 2002; Laycock et al., 2012; Pina Rodrigues et al., 2017b) and, in an important recent study, it has been demonstrated a causal link between magnocellular deficits and DD (Gori et al., 2016). Hari and Renvall (2001) proposed that parietal attentional dysfunction could underlie such deficits. Specifically, these authors suggested sluggish attentional shifting (SAS) as a causal factor for temporal processing impairment in DD (Hari and Renvall, 2001). Attentional shifting refers to the engagement mechanisms onto a relevant object and subsequent disengagement from the previous object to the next one. In terms of reading processes, this failure can cause impaired speech segmentation and scanning of letter strings, which in turn can result in poor phonemic/graphemic representations and, thus, in reading difficulties (Lallier et al., 2010; Krause, 2015). Another brain structure that has been implicated either in exogenous attention orienting mechanisms as in reading impairments in DD is the cerebellum. Besides the evidence that oculomotor structures in the cerebellum are involved in the generation of exogenous shifts of attention (Baier et al., 2010; Striemer et al., 2015b) and that other cerebellar structures may provide input to the exogenous attention neural network (Striemer et al., 2015a), it has been proposed that cerebellum abnormalities in DD can lead to an impairment in skill automatization with consequent reading difficulties (Nicolson et al., 2001; Nicolson and Fawcett, 2005).

Several studies have shown that automatic exogenous orientation of attention is impaired in dyslexic children (see Facoetti, 2012; Gabrieli and Norton, 2012 for reviews). This subject was particularly explored by Facoetti and colleagues in a series of experiments (Facoetti et al., 2000b, 2003a, b, 2005, 2010; Facoetti and Molteni, 2001; Ruffino et al., 2014). By using cueing paradigms, in which participants are asked to react as quickly as possible to the appearance of target stimuli preceded by spatial cues, and manipulating the stimulus onset asynchrony (SOA) (i.e., interval between cue and target-stimulus) to activate both endogenous and exogenous systems, these authors showed that cueing effects are absent in dyslexics only at the shortest intervals, i.e., when exogenous mechanism are recruited (Facoetti et al., 2000b, 2003a). This impairment was found to be correlated with sublexical reading deficits in children with DD, pointing to a direct link between phonological skills and exogenous attentional mechanisms (Facoetti et al., 2010; Ruffino et al., 2014, 2010). Importantly, Franceschini et al. (2012) found, in a longitudinal study, that prereading exogenous attention orienting, assessed by cueing paradigms, predicts reading acquisition and several reading skills, such as text, word, and pseudoword reading. These authors found that the abnormality in orienting of attention is rather prevalent early in development. In their sample, 60% of future poor reader children were impaired in attention orienting at the prereading stage. Nevertheless, the role of attentional orienting mechanisms in the reading deficits is a subject still under debate. Several studies also using cueing paradigms with variable SOAs suggested preserved exogenous and endogenous attention orienting in adults with DD (Judge et al., 2007, 2013; Moores et al., 2011, 2015), raising the hypothesis that deficits in exogenous orienting of attention observed in DD children do not persist and hindering the claim of a causal link between such deficits and reading impairments.

The literature concerning spatial distribution of visual attention in DD is also contradictory. While some studies found an abnormal spatial distribution in these patients (Geiger and Lettvin, 1987; Geiger et al., 1992, 2008; Facoetti and Turatto, 2000; Facoetti and Molteni, 2001), others did not (Judge et al., 2007; Moores et al., 2015). Among the studies that favor the atypical spatial distribution hypothesis are the ones from Geiger and Lettvin (1987) and Geiger et al. (1992, 2008), who found that, in the presence of lateral masking, dyslexics recognize letters visually farther in the periphery than typical readers. The authors suggested that dyslexics exhibited a wider visual perceptual mode. Their finding was corroborated by other studies (Perry et al., 1989; Dautrich, 1993; Lorusso et al., 2004) and found to be present across different subtypes of DD (Lorusso et al., 2004). Additionally, Facoetti et al. (2001) and Facoetti and Molteni (2001) studied attention orienting at different visual eccentricities and found that DD children did not show normal eccentricity effects as controls, corroborating a diffuse-distributed attention mode in DD. On the other hand, Judge et al., using the task used by Facoetti and Molteni (2001), showed that, unlike children, DD adults exhibit normal eccentricity effects (Judge et al., 2007). Their work was supported by a study (Moores et al., 2015) in which results show similar effects of eccentricity and cueing in DD and controls also arguing against the notion of a more distributed attention in DD adults than in typical readers.

Taking into account the literature discrepancies and the ongoing debate described above, the main aim of the present study was to investigate exogenous visual attention in DD adults and its peripheral spatial distribution. Particularly, we intended to investigate facilitation and inhibition attentional effects in DD adults and controls. Facilitation effect refers to the fact that when a target is preceded by a spatial cue at the same location, it’s detection is faster than at uncued locations due to the shifting of attention to the cued location prior to the presentation of the target. On the other hand, attentional inhibition refers to the ability to suppress and ignore salient yet irrelevant features in the scene (Posner, 1980). To assess exogenous orienting of attention we used a classical cueing paradigm, in which peripheral pre-cues were presented, followed by a short SOA. We then adapted this paradigm to a discrimination task. Discrimination requires more attentional resources than simple detection and, therefore, is expected to be more prone to cueing effects. In order to study attentional effects, and since automatic orienting is supposed to occur regardless of the validity of the cue or even when subjects are not aware of the cue (McCormick, 1997; Rosen et al., 1999), uninformative cues were included in the experiment. Spatial distribution of attention was tested by presenting the target stimuli at parafoveal and perifoveal peripheral eccentricities, ranging from 8° to 14°.

## Materials and Methods

### Participants

Eighteen developmental dyslexics and nineteen age and IQ matched controls were recruited. Individuals with dyslexia had all received a formal diagnosis of dyslexia from a qualified psychologist or an education authority official, and none had been diagnosed with any other developmental disorder (e.g., ADHD) or any neurological or psychiatric disorder. Controls were adults with no history of learning, developmental, cognitive, neurological, or neuropsychiatric disorders. All participants were assessed in terms of reading performance and intelligence level. For the reading assessment, a sub-test from the Psycholinguist Assessments of Language Processing in Aphasia - Portuguese version (PALPA-P; Castro et al., 2007) was used. In this sub-test, participants were asked to read a list of 60 words and pseudowords as quickly as possible. The measures obtained from this sub-test were reading speed (in seconds) and accuracy (number of words correctly read). Intelligence level was measured through the Raven Progressive Matrices Test – Set 1 (RPM; Raven et al., 1976). All participants had normal or corrected to normal vision. Participants’ demographics and reading and intelligence scores are summarized in Table 1. The study was conducted in accordance with the tenets of the Declaration of Helsinki and was approved by the Ethics Committee of the Faculty of Medicine of the University of Coimbra. Written informed consent was obtained from the participants, after an explanation of the nature of the study.

**TABLE 1:** Summary statistics for the two groups of participants.

	Dyslexics (*n* = 18)			Controls (*n* = 19)			
	Mean	Range	SD	Mean	Range	SD	*p*-Value
Age (years)	27.08	19–44	7.05	25.05	20–36	4.03	0.443
Education (years)	15.56	13–17	1.58	16.21	14–17	1.08	0.149
RPM	10.08	8–12	1.19	11.14	8–12	1.68	0.063
PALPA-P reading speed (s)	71.67	42–105	18.20	42.33	31–52	7.43	<0.05
PALPA-P accuracy	50.31	42–57	4.48	57.67	56–59	1.21	<0.01
Gender (m:f)	8:10			9:10			1.00

*P-values for t-test comparisons (except for gender, for which the Chi-square test was used) between the two groups are reported (p < 0.05 values are considered significant).*

### Apparatus

The experiment was conducted in a dark room. Stimuli were delivered using the Presentation software (Neurobehavioral Systems) on a 38 × 30.2 cm (41.6 × 33.6° visual angle) computer screen with a resolution of 1280 × 1024 pixels and a luminance of 108 cd/m2. The distance between the subjects’ eyes and the computer screen was 52 cm. A chin and forehead rest was used to ensure a stable viewing position throughout testing. To ensure that subjects fixated the center of the stimulus display during the experiment, the subject’s gaze position was monitored using an eye-tracker SMI iViewX High-speed (SensoMotoric Instruments GmbH, Germany).

### Stimuli and Procedure

The stimuli consisted of Gabor patches, comprising a simple sinusoidal grating convolved by a Gaussian envelope (spatial frequency – 2 cpd; envelope SD – 0.25°; contrast – 50% Michelson). Stimuli were presented one at a time at two levels of viewing eccentricity (parafoveal and perifoveal) in the four quadrants of the visual field. In the parafoveal level, stimuli appeared at 8 and 10 degrees of eccentricity, while in the perifoveal level appeared at 12 and 14 degrees (Strasburger et al., 2011). The patches were randomly oriented at 45 or 135 degrees from the vertical and participants were asked to discriminate, as quickly as possible, the orientation of the gratings by pressing the corresponding button of a response box. A fixation cross was presented at the center of the screen and participants were instructed to fixate the cross throughout the whole experiment. Participants’ reliability was evaluated by randomly interleaving false positive and false negative catch trials. In the false negative trials stimuli were presented at the center of the screen, in the location where subjects were instructed to fixate. False positive trials consisted in trials where only the pre-cue was presented. In these trials participants were instructed to not respond. Performance reliability was assessed by monitoring fixation loss and computing false positive and negative errors. A percentage of ≥33% of false positive and negative errors was defined as exclusion criteria, according to standard procedures (Ribeiro et al., 2012; Mateus et al., 2013). The sizes of the stimuli were scaled with viewing eccentricity using a magnification factor estimate for the temporal visual field, M (Rovamo and Virsu, 1979): *M* = M0(1 + 0.29E + 0.000012E3), where E represents eccentricity and M0 represents the size of the stimuli at the smallest eccentricity. The smallest eccentricity in our experiment was the fovea where the stimulus size was 0.83°. Therefore, stimulus sizes were 2.76°, 3.24°, 3.74°, and 4.2° for the 8°, 10°, 12°, and 14° of eccentricity, respectively.

Exogenous orienting of attention was assessed using a variant of Posner’s task (Posner, 1980) comprising visual targets preceded by spatial cues (valid, invalid, and neutral). In the valid and invalid trials, the cue consisted in a salient black dot (0.23°) presented either at the same eccentricity and visual quadrant of the subsequent stimuli (valid) or at the same eccentricity but at a randomized different visual quadrant of the subsequent stimuli (invalid). In the neutral trials, four identical black dots were presented simultaneous at 14° of eccentricity in the four visual quadrants. Attentional facilitation effects were obtained computing the difference between neutral and valid cue conditions while attentional inhibition effects referred to the difference between invalid and neutral cue conditions. Participants were informed of the possible appearance of black dots in the screen and were instructed to not attend to them.

**FIGURE 1:**
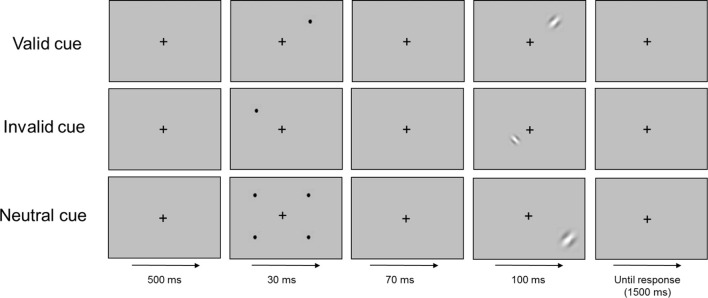
Schematic representation of the time course (left to right) of the procedure.

Each trial began after subjects continuously foveated the fixation cross for 500 ms. After that, the cue was presented for 30 ms, followed by a stimulus onset asynchrony (SOA) of 70 ms, after which the stimulus appeared for 100 ms. The maximum time allowed for response was 1500 ms (see Figure 1).

The experiment consisted of 2 runs of 600 trials each (1200 in total), separated by an interval in which the subjects were allowed to rest. Eye-tracker calibration was repeated after the rest period. Stimuli were randomly presented in six conditions (two levels of eccentricity × three types of cue), each having 160 trials. In addition, 120 false positive and 120 false negative trials were presented. Therefore, the experiment consisted on 1200 trials, divided in 960 experimental trials and 240 control trials, with a maximum duration of 1 h for each participant (45 min for the experiment and 15 min for rest and recalibration). Before the experiment began, participants made a practice run (80 trials) to become familiarized with the task. The dependent variable of interest was the response time (RT) since we expected accuracy to be close to ceiling.

### Statistical Analysis

All statistical analyses were performed using the IBM SPSS statistical software package, version 20.0 (SPSS, Inc., Chicago, IL, United States). Mean correct RTs were analyzed with a mixed ANOVA, with group as the between factor (dyslexics and controls) and eccentricity (8°, 10°, 12°, and 14°) and cue (valid, invalid, and neutral) as within factors. Results with *p* < 0.05 were considered statistically significant. Outliers, defined as RTs above or below 3 SD from the group’s mean, were not detected. None of the participants scored ≥33% in false positive and false negative trials and, therefore, all participants were included in the analysis.

## Results

As expected, accuracy was close to ceiling, being above 90% in all conditions in both groups, which ensured that both dyslexics and controls were able to perform the task correctly (Table 2). There was no significant main effect of group, as well as no significant effect of cue. Both dyslexics and controls had similar accuracy across the task and the type of cue did not affect participant’s accuracy. The main effect of eccentricity was significant [*F*(1,35) = 4.24, *p* < 0.05; $\eta_{\text{p}}^{2}=0.114$]. Participants were more accurate at parafoveal eccentricities than at perifoveal (difference = 0.7%, *p* < 0.05). There were no significant interactions.

**TABLE 2:** Means and standard deviation (in brackets) of the hit rates (percentage) of dyslexics and controls at the four viewing eccentricities (8°, 10°, 12°, and 14°) and for the three cue types (valid, invalid, and neutral).

	Dyslexics			Controls		
	Valid	Invalid	Neutral	Valid	Invalid	Neutral
8°	92.81 (3.52)	92.42 (6.40)	93.44 (4.97)	94.61 (5.32)	95.13 (5.53)	93.82 (5.84)
10°	93.28 (4.74)	92.81 (4.80)	89.53 (7.27)	93.75 (4.86)	93.62 (5.03)	93.75 (6.24)
12°	90.55 (5.83)	92.19 (4.29)	92.42 (4.67)	95.53 (4.32)	93.42 (5.73)	94.54 (5.91)
14°	91.56 (6.10)	89.67 (5.23)	89.68 (6.17)	94.34 (4.48)	93.75 (4.23)	93.03 (6.32)

**FIGURE 2:**
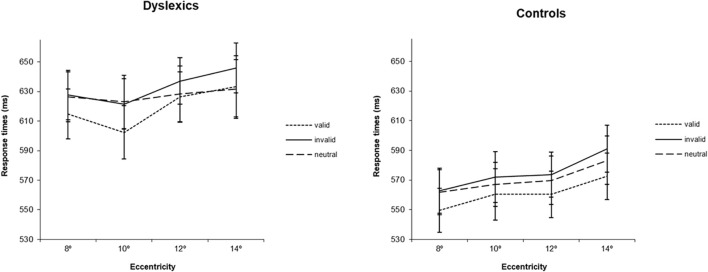
Average response times (ms) for the three types of cues (valid, invalid and neutral) at four eccentricities (8°, 10°, 12°, 14°) for dyslexics and controls. Error bars indicate ±SE.

Regarding RTs analysis, the main effect of group was significant [*F*(1,35) = 6.41, *p* < 0.05; $\eta_{\text{p}}^{2}=0.155$] showing that dyslexics were globally slower than controls (RTs were 626 ms for DD and 570 ms for controls). The main effect of cue was also significant [*F*(2,70) = 13.13, *p* < 0.001; $\eta_{\text{p}}^{2}=0.273$], and similar in both groups [*F*(2,70) = 0.04, *p* = 0.958]. Participants were faster when a valid cue was presented than when invalid (*p* < 0.001) or neutral (*p* < 0.01) cues were displayed. Eccentricity was also found to have an effect in RTs [*F*(3,105) = 19.06, *p* < 0.001; $\eta_{\text{p}}^{2}=0.353$]. Overall, participants became slower with increases in eccentricity, except between 8° and 10° where the RTs were equivalent. The smallest (but still significant) difference between eccentricities was found for the comparison between 10° and 12° (difference = 8 ms, *p* < 0.05) (see Figure 2).

**FIGURE 3:**
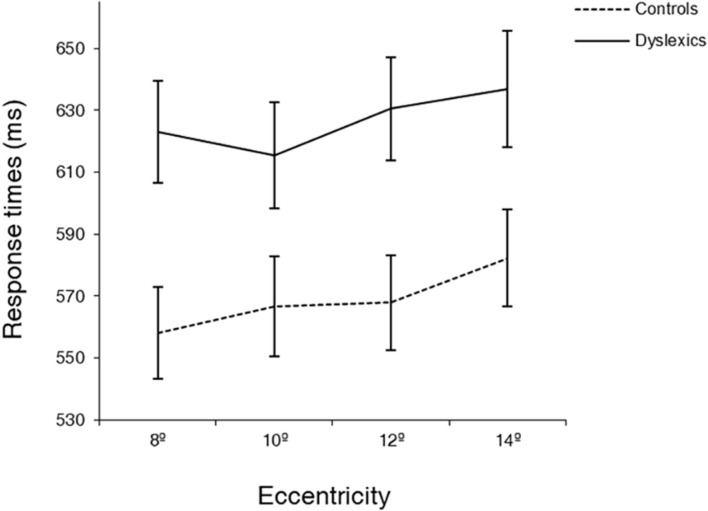
Average response times (ms) at four eccentricities (8, 10, 12, 14°) for dyslexics and controls. Note the different behavior of dyslexics and controls at 10° of eccentricity. Error bars indicate ±SE.

Interestingly, the eccentricity × group interaction was found to be significant [*F*(3,105) = 3.12, *p* < 0.05; $\eta_{\text{p}}^{2}=0.082$], showing that eccentricity had a different effect on the RTs of each group (see Figure 3). *Post hoc* analysis for the different pairs of eccentricity revealed a different behavior of dyslexics at 10° of eccentricity [*F*(1,35) = 11.38, *p* < 0.01; $\eta_{\text{p}}^{2}=0.245$ for the comparison between 8° and 10° and *F*(1,35) = 6.25, *p* < 0.05; $\eta_{\text{p}}^{2}=0.152$ for the comparison between 10° and 12°]. While the RTs of controls followed the expected increase with eccentricity, dyslexics showed an inflection at 10° of eccentricity, increasing again at 12°. Additionally, to further investigate the effect of eccentricity in each group, one-way ANOVAs were performed in each group separately, using eccentricity (8°, 10°, 12°, 14°) as within factor. Controls showed a trend for a significant difference between 8° and 10° eccentricity (difference = 9 ms, *p* = 0.06), as well a significant difference between 12° and 14° eccentricity (difference = 14 ms, *p* < 0.001). On the contrary, DD participants only showed a significant difference between 10° and 12° eccentricity (difference = 15 ms, *p* < 0.05). Importantly, controls were, as expected, faster at 8° of eccentricity than at 14° of eccentricity (difference = 27 ms, *p* < 0.001). In contrast, DD adults showed no significantly different RTs at the most distant eccentricities tested.

**FIGURE 4:**
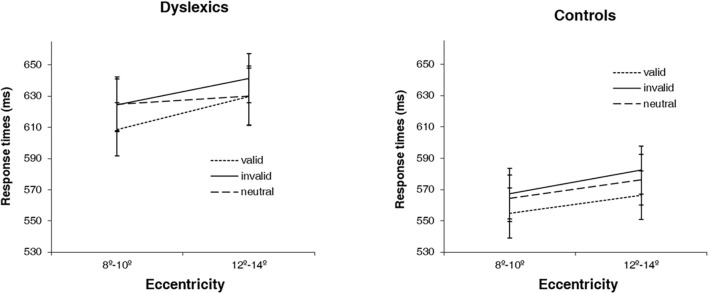
Average response times (ms) for the 3 types of cues (valid, invalid and neutral) at two levels of eccentricity (8°–10° and 12°–14°) for dyslexics and controls. Error bars indicate ±SE.

The non-linear behavior of DD participants, particularly at 10° of eccentricity, as well as the different distribution of the data in both groups (see Figure 2), motivated us to explore the effect of cue at different levels of eccentricities. Based on the different pattern of behavior in dyslexics that we observed at 10° of eccentricity, we therefore defined this eccentricity as a cutoff and collapsed the 4° of eccentricity in two levels, the first comprising 8° and 10° (equal or below the identified 10° cutoff); and the second comprising 12 and 14° (above the cutoff). We then investigated attentional facilitation (difference between neutral and valid conditions) and attentional inhibition (difference between invalid and neutral conditions) effects at near (8°–10°) and far (12°–14°) periphery. For that, we performed a mixed ANOVA, with group as between factor (dyslexics and controls) and eccentricity (8°–10° and 12°–14°) and attentional effect (facilitation and inhibition) as within factors. Notably, we found a significant group × eccentricity × attentional effect interaction [*F*(1,35) = 4.24, *p* < 0.05; $\eta_{\text{p}}^{2}=0.114$]. *Post hoc* analyses showed that both groups have a different facilitation effect depending on the spatial location of the stimuli [*F*(1,35) = 4.42, *p* < 0.05; $\eta_{\text{p}}^{2}=0.118$]. While in controls, the facilitation effect is similar at near and far periphery (mean 8/10 = 9.44, mean 10/12 = 9.94 ms; difference = 0.50 ms, *p* > 0.05), in dyslexics it is absent at the far periphery (mean 8/10 = 15.91, mean 10/12 = −4.04 ms; difference = 19.95 ms, *p* < 0.05) (see Figure 4).

## Discussion

In the current study, we investigated the exogenous orienting of attention and its spatial distribution across the peripheral visual field in dyslexic and typically reading adults.

We showed that dyslexic adults have temporal deficits in orientation of attention. Although this has already been described in children with DD (Facoetti et al., 2000b, 2003a, b, 2005; Banfi et al., 2017), the current study is, to our knowledge, the first to demonstrate this impairment in adults with DD. Our finding thus indicates that the temporal deficits in orientation of attention in this population are not restricted to childhood and, therefore, persist into adulthood. Our study contrasts with other works (Judge et al., 2007, 2013; Moores et al., 2015), which found similar RTs in adults with DD and controls in tasks requiring rapid orientation of attention. This discrepant result can be accounted in terms of spatial position of the stimuli. In these studies, the eccentricities at which the stimuli were presented ranged from 0.7° (Moores et al., 2015) to 9° (Judge et al., 2007) of visual angle. The eccentricities tested in the current study were substantially larger, with a minimum of 8° and a maximum of 14°. If DD patients suffer from anomalous peripheral spatial distribution of attention, this fact can by itself indicate that this discrepancy is only apparent, and may be due to the herein proposed distinct structure of spatial attention.

Actually, the hypothesis of abnormal spatial distribution of attention in the peripheral visual field of DD adults is supported by the two additional main findings of this study. First, DD adults showed abnormal eccentricity effects, reflecting a wider distribution of attention than controls. Such pattern has been already described in children with DD (Facoetti et al., 2000a). However, previous studies (Judge et al., 2007; Moores et al., 2015) have found similar effects of eccentricity in adults with and without DD. Nonetheless, the eccentricities tested corresponded to foveal, parafoveal, and perifoveal processing, while the present study used more peripheral eccentricities, outside of the macular zone (Strasburger et al., 2011). Our finding, therefore, adds to previous evidence by showing that the abnormal distribution of peripheral visual attention observed is present in adults thereby persisting beyond development.

The second finding that supports an atypical spatial distribution of visual attention in DD adults is that attentional cueing effects in DD are dependent on viewing eccentricity. In accordance with previous studies (Posner, 1980; Posner et al., 1980), normal reading adults showed cue effects at all levels of eccentricity. On the contrary, RTs of dyslexic adults could only benefit from valid cues (i.e., show facilitation effects) when stimuli were presented at less peripheral eccentricities. Thus, DD adults are not capable of efficiently using valid cues to rapidly direct attention to more peripheral eccentricities, suggesting an attentional engagement deficit at far periphery (Posner et al., 1984). This result is in accordance with that of Moores et al. (2015) who found an indication that DD adults need more time to focus attention to far eccentricities. However, it is important to note that Roach and Hogben (2004) found a similar impairment at lower eccentricities. Nonetheless, their task included distractor stimuli in set sizes up to 16 elements, which likely brought an increment of difficulty to DD adults since it is known that crowding affects DD more than controls (Moores et al., 2011).

It is important to note that reading experience can influence perceptual and cognitive functions, also in adult brains (Dehaene et al., 2015). However, given the fairly high reading experience of our dyslexic sample (mean years of education/instruction above 15 years, at least university attendance), it is very unlikely that the present results are merely consequence of reduced reading exposition in dyslexic group.

Along with attention impairments, phonological awareness (e.g., Snowling, 1981) and automatization (Nicolson et al., 2001) deficits are known to be also present in DD individuals. Interestingly, on one hand, since orienting of attention is crucial to the selection and segmentation of stimuli, deficits on this mechanism may precede the difficulties of dyslexics on the perception and manipulation of phonemes. On the other hand, given the automatic nature of the orienting deficits found in this study, such deficits are consistent with the automatization deficits also found in this condition.

Our findings are also consistent with the notion that covert attention mechanisms, as measured by Posner-like paradigms, operate in a distinct manner in central and peripheral vision in health and disease, as also observed in a previous study from our group in Parkinson disease (Sampaio et al., 2011). In that study we found impaired high-level attentional modulation of contrast sensitivity in the visual periphery (up to 15°), where mechanisms of covert attention are at higher demands. A critical role for peripheral vision is justified by the fact that it can be used to make a snapshot of the local context (van Asselen and Castelo-Branco, 2009).

A limitation of the present study refers to the lack of an assessment of attention with a conventional attention test, such as the d2 test (Brickenkamp and Zillmer, 1998). However, although one may expect a relationship between results on conventional attention tests and on the task performed in this study, the specific mechanism of attention targeted in this work (exogenous orienting of attention) is not covered by such tests. The characteristics of this mechanism (involuntary, automatic, rapid, and stimulus-driven) hinders its assessment by conventional attention tests and, from our knowledge, there are no commercial tests developed to evaluate it. Nonetheless, one may expect significant correlations between results on the experimental task and on conventional attention tests, due to the involvement in both cases of processes such as sustained attention and processing and perceptual speed, that are assessed on classical and widely used attention tests.

Finally, we speculate that our results may be interpreted within the framework of the role of right posterior parietal cortex in spatial attention. Particularly, the right temporo-parietal junction (TPJ) is known to be involved in the network responsible for exogenous orienting of attention (Corbetta and Shulman, 2002, 2011). Consistent with the hypothesis of a right posterior parietal dysfunction in dyslexia (e.g., Hari et al., 1999; Facoetti et al., 2001), some studies observed deficient activations in the right TPJ in dyslexics when performing phonological decoding tasks (e.g., Hoeft et al., 2006). Moreover, a very recent study (Lazzaro et al., 2021) has shown significant effects of tDCS on temporo-parietal regions either on reading performance as on visuo-spatial skills of dyslexic children and adolescents. Overall, the findings of the present study endorse this hypothesis by showing that the mechanisms of rapid orienting of spatial attention are impaired in adults with DD.

## Conclusion

In the present study we found that adults with dyslexia exhibit global temporal deficits in a task requiring orientation of attention. Moreover, we showed that an abnormal peripheral spatial distribution of attention is also not restricted to children with dyslexia and persists into adulthood. Overall, our results suggest an impairment of the neural network underlying selective spatial attention (rooted at right posterior parietal regions) in dyslexia.

## Data Availability Statement

The raw data supporting the conclusions of this article will be made available by the authors, without undue reservation.

## Ethics Statement

The studies involving human participants were reviewed and approved by Ethics Committee of the Faculty of Medicine of the University of Coimbra. The patients/participants provided their written informed consent to participate in this study.

## Author Contributions

AP, MC-B, and MA designed the study. AP collected and analyzed the data. All authors discussed the results. AP wrote the manuscript with input from MC-B and MA.

## Conflict of Interest

The authors declare that the research was conducted in the absence of any commercial or financial relationships that could be construed as a potential conflict of interest.

## Publisher’s Note

All claims expressed in this article are solely those of the authors and do not necessarily represent those of their affiliated organizations, or those of the publisher, the editors and the reviewers. Any product that may be evaluated in this article, or claim that may be made by its manufacturer, is not guaranteed or endorsed by the publisher.
